# Wearable Inertial Sensors for Gait Analysis in Adults with Osteoarthritis—A Scoping Review

**DOI:** 10.3390/s20247143

**Published:** 2020-12-13

**Authors:** Dylan Kobsar, Zaryan Masood, Heba Khan, Noha Khalil, Marium Yossri Kiwan, Sarah Ridd, Matthew Tobis

**Affiliations:** 1 Department of Kinesiology, Faculty of Science, McMaster University, Hamilton, ON L8S 4L8, Canada; masooz1@mcmaster.ca (Z.M.); khanh31@mcmaster.ca (H.K.); khalin6@mcmaster.ca (N.K.); kiwanm@mcmaster.ca (M.Y.K.); tobism1@mcmaster.ca (M.T.); 2 Department of Psychology, Neuroscience, and Behaviour, Faculty of Science, McMaster University, Hamilton, ON L8S 4L8, Canada; ridds@mcmaster.ca

**Keywords:** inertial sensors, wearable technology, osteoarthritis, gait, biomechanics, walking, review

## Abstract

Our objective was to conduct a scoping review which summarizes the growing body of literature using wearable inertial sensors for gait analysis in lower limb osteoarthritis. We searched six databases using predetermined search terms which highlighted the broad areas of inertial sensors, gait, and osteoarthritis. Two authors independently conducted title and abstract reviews, followed by two authors independently completing full-text screenings. Study quality was also assessed by two independent raters and data were extracted by one reviewer in areas such as study design, osteoarthritis sample, protocols, and inertial sensor outcomes. A total of 72 articles were included, which studied the gait of 2159 adults with osteoarthritis (OA) using inertial sensors. The most common location of OA studied was the knee (*n* = 46), followed by the hip (*n* = 22), and the ankle (*n* = 7). The back (*n* = 41) and the shank (*n* = 40) were the most common placements for inertial sensors. The three most prevalent biomechanical outcomes studied were: mean spatiotemporal parameters (*n* = 45), segment or joint angles (*n* = 33), and linear acceleration magnitudes (*n* = 22). Our findings demonstrate exceptional growth in this field in the last 5 years. Nevertheless, there remains a need for more longitudinal study designs, patient-specific models, free-living assessments, and a push for “Code Reuse” to maximize the unique capabilities of these devices and ultimately improve how we diagnose and treat this debilitating disease.

## 1. Introduction

Osteoarthritis (OA) is a degenerative joint disease characterized by the loss of cartilage and changes in bone, resulting in pain, disability, and reduced quality of life. It is the most prevalent musculoskeletal disease, with conservative estimates suggesting that it affects approximately 20% of adults [1,2]. Moreover, it is one of the leading causes of physical disability in the world and given our aging population, its global burden is expected to continue expanding [3].

While there is no cure for OA, lower limb biomechanical data collected during walking can provide valuable information on the etiology, progression, and treatment of this disease [4,5,6,7]. Specifically, joint kinetics offer a proxy to the loading environment within the hip [8] or knee [9]. Most notably, an increased knee adduction moment (KAM) has been linked to the structural progression of knee OA [10,11,12]. In addition to joint kinetics, spatiotemporal (ST) parameters (e.g., stride times, cadence, etc.) and joint kinematics (e.g., joint angles, joint range of motion, etc.) are also commonly used to study OA [9]. Unfortunately, these biomechanical outcomes often require a gait analysis laboratory which utilizes optical motion capture cameras and force plate systems. While such systems offer exceptional accuracy and reliability, they are expensive and time-consuming which makes them inaccessible to many clinicians. Furthermore, the protocols required for these gait analyses can make them a poor representation of real-world gait patterns [13].

Fortunately, the advent of wearable inertial sensors has provided an accessible and affordable alternative to conventional optical gait analysis systems [14]. Wearable inertial sensors, or inertial measurement units, measure motion (e.g., linear accelerations, angular velocities) using one or more individual sensors (e.g., accelerometer, gyroscope, magnetometer). By securely attaching inertial sensors to various segments of the body, a variety of biomechanical outcomes can be obtained with similar validity and reliability to optical systems [15]. Furthermore, inertial sensor data collections are not tethered to conventional gait analysis laboratories. Therefore, it is not surprising that the use of inertial sensors to study OA biomechanics has exploded in popularity. This growth was highlighted in a scoping review by Small et al., 2019 [16], which examined the use of inertial sensors to assess the outcomes following knee arthroplasty. While this was an important summary of all inertial sensor research related to knee arthroplasty (e.g., gait analysis, functional assessments, physical activity measures, etc.), there remains a large body of fragmented literature utilizing inertial sensors across the entire field of OA gait biomechanics research.

Therefore, in conducting this scoping review, we aimed to summarize the growing body of literature using wearable inertial sensors for gait analysis in OA. Specifically, our objective was to identify biomechanical outcomes and applications of wearable inertial sensors for assessing walking gait in adults diagnosed with lower limb OA. We aimed to highlight the quality and types of research prominent in this field, with a focus on biomechanical outcomes and important gaps to be addressed in future research.

## 2. Methods

Our scoping review was conducted following the Preferred Reporting Items for Systematic Reviews and Meta-Analyses (PRISMA) guidelines. This format was used to provide a broader perspective on the use of inertial sensors across the field of OA gait biomechanics and to identify important gaps in the literature [17]. In doing so, we provide an update on the use of inertial sensors for gait biomechanics research in OA surgical interventions previously examined by Small et al. [16]. More importantly however, we expand this review into all other areas of OA gait research previously unexamined in any review.

### 2.1. Eligibility Criteria

Studies were included within this scoping review if they used inertial sensors (i.e., accelerometers, gyroscopes, and/or magnetometers) for walking gait analysis in adults diagnosed with lower limb OA (e.g., hip, knee, or ankle). We considered any analysis of walking patterns which involved (i) ST parameters (e.g., step time, step length, stride time, etc.), (ii) kinematics (e.g., segment orientations or joint angles), (iii) kinetics (e.g., joint moments), or (iv) other inertial sensor measures (e.g., frequency analysis, impact accelerations, waveform analysis, etc.). However, other measures that do not provide an assessment of walking gait patterns (e.g., daily step count, physical activity, energy expenditure, etc.) were not considered. Therefore, any study only examining such outcomes were not eligible. Additionally, conference abstracts, systematic reviews, and meta-analyses were also excluded. A complete list of the inclusion and exclusion screening criteria can be found in Appendix A.

### 2.2. Search Strategy and Screening

We conducted our literature search using the following six databases: MEDLINE, EMBASE, CINAHL, SPORTDiscus, Web of Science Core Collection, and Engineering Village. An individualized search strategy was developed for each database to optimize the search scope using the three broad topics of inertial sensors, gait, and OA. The full search strategy syntax for each database can be found in Appendix B.

Our search was conducted on 9 June 2020 and all studies identified were imported to Covidence systematic review software for screening. Duplicates were automatically identified by Covidence and manually verified by a reviewer (D.K.) before their removal. Title and abstract screening were conducted by two independent reviewers (M.Y.K. and S.R.) through the Covidence software based on the screening criteria (Appendix A). Any conflicts were resolved through discussion between the two reviewers, with a third reviewer (D.K.) to resolve discrepancies when a consensus could not be reached. Full-text screening was subsequently carried out by two independent reviewers (M.Y.K. and M.T.) to determine the final review inclusion. Discrepancies regarding any decision to include or reason to exclude were discussed between reviewers, with a third reviewer (D.K.) to consult if an agreement could not be reached.

### 2.3. Methodological Quality

A quality assessment for each study was conducted independently by two raters (N.K. and S.R.) using a modified version of the Critical Appraisal of Study Design for Psychometric Articles [18]. This guide comprised of twelve questions to be assessed in five areas related to the study question, study design, measurements, analyses, and recommendations (See Appendix C). Each item was scored from 0 to 2, with “0” being unsatisfied, “1” being adequately satisfied, and “2” being completely satisfied. Thus, a total score out of 24 could be awarded and converted to a percentage. The two raters independently evaluated the study quality in blocks of 10, before virtually meeting to determine a consolidated score for each study. Based on this consolidated score, studies were classified as high quality (>85%), moderate quality (70–84.9%), low quality (50–69.9%), and very low quality (<50%). An intraclass correlation coefficient (ICC 3, k) was calculated to determine the inter-rater reliability of pre-consensus scores. Overall, this quality assessment scoring was conducted to provide an overview of the methodological quality in this area of research and support our recommendations from individual studies.

### 2.4. Data Extraction

Following the full-text screening and inclusion of studies, data were extracted by a primary reviewer (H.K.) and verified for accuracy by two additional reviewers (N.K. and Z.M.). Data extraction occurred in two broad areas: study characteristics and inertial sensor protocol. The study characteristics consisted of the OA sample, study design, and additional characteristics relating to the publication itself (e.g., open access, supplementary data, etc.). The inertial sensor protocol extracted consisted of sensor models, specifications, placement, data collection setting (e.g., in-lab, out-of-lab, free-living), walk length, and biomechanical outcomes. Lastly, we identified studies using machine learning algorithms in their analysis as those consisting of training data for model development and testing data for model validation (e.g., leave-one-out, cross-validation, test set, etc.). These criteria were determined to minimize ambiguity in objectively defining machine learning amidst the wide range of possible algorithms and statistical analyses in this area.

## 3. Results

### 3.1. Search Results and Screening

**Figure 1:**
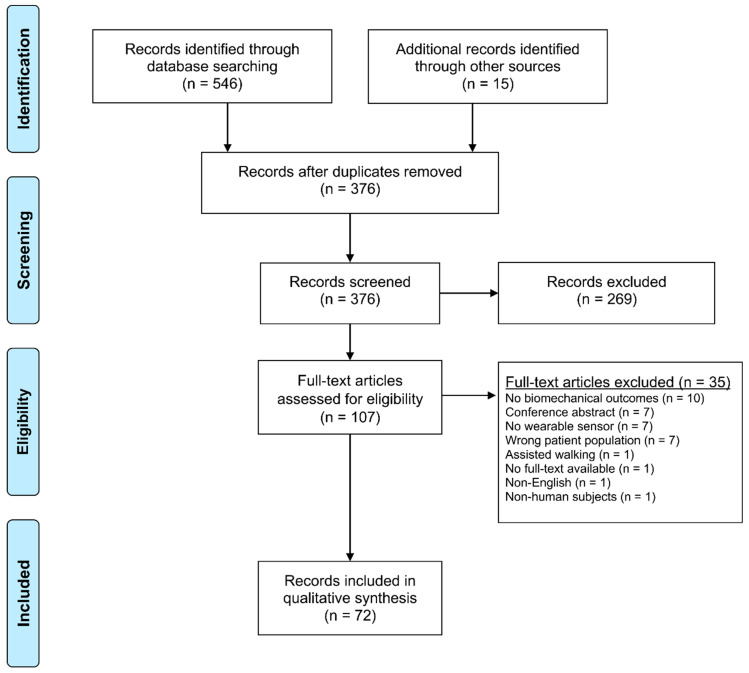
Preferred Reporting Items for Systematic Reviews and Meta-Analyses (PRISMA) flow diagram.

Our search strategy identified a total of 561 articles. The duplicate removal rendered 376 articles, which were screened at the title/abstract level. A full-text screening process of 107 articles resulted in 72 articles, and these 72 articles were included in our scoping review [19,20,21,22,23,24,25,26,27,28,29,30,31,32,33,34,35,36,37,38,39,40,41,42,43,44,45,46,47,48,49,50,51,52,53,54,55,56,57,58,59,60,61,62,63,64,65,66,67,68,69,70,71,72,73,74,75,76,77,78,79,80,81,82,83,84,85,86,87,88,89,90]. The PRISMA flow diagram presented in Figure 1 documents all exclusions at the full-text level. The most common reason for exclusion at the full-text level was “No biomechanical outcomes”, which was generally the result of studies measuring only physical activity or step count data.

### 3.2. Quality Assessment

Our quality assessment did not find any studies which were rated as high quality, but rather identified most studies as low (*n* = 43) or moderate quality (*n* = 24). Articles generally scored the highest on the background and research question (Q1) and sensor information (Q7), but the lowest on the sample size justification (Q5). Agreement between raters was found to be moderate with an ICC (3, k) of 0.74 (95% CI 0.58–0.84). Consolidated quality rating for all included studies can be found in Table 1.

### 3.3. Osteoarthritis Sample Demographics

The gait of 2159 adults with OA were examined using wearable technology across the 72 studies. The most common location of OA studied was the knee (*n* = 46), followed by the hip (*n* = 22), and the ankle (*n* = 7). Additional healthy controls were present for comparison in 67% of these studies. The weighted average for the age, body mass index (BMI), and percentage of females in the OA cohorts was 66 (range: 44–74) years old, 27 (range: 22–33) kg/m^2^, and 57% (range: 0–100) female, respectively. See Table 2 and Supplementary Table S1 for a list of sample demographics.

### 3.4. Study Designs

**Figure 2:**
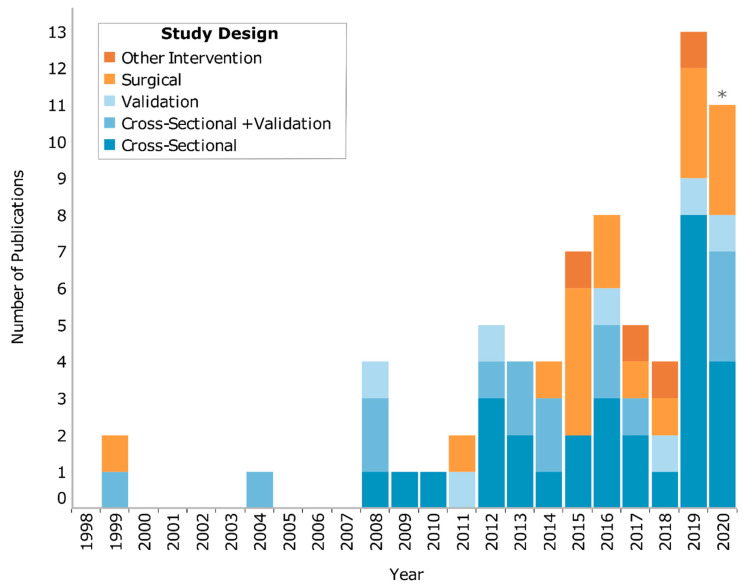
Number of included studies published each year, grouped by study (* up to June 2020). An interactive version of this figure which highlights the referenced studies can be found at: https://public.tableau.com/profile/dylan.kobsar#!/vizhome/WearableOAReview_Story/Fig2_Fig3_Story.

Study designs were grouped into longitudinal, cross-sectional, and validity/reliability. The most common study design was found to be cross-sectional (*n* = 44), with most examining the differences in gait between OA and controls (*n* = 34). There were 21 studies that utilized some type of longitudinal design, with most examining the effect of surgery (*n* = 17) and four examining other interventions (e.g., exercise, gait retraining). See Figure 2 for a visualization of the study designs by year. Additionally, 44% of publications were open access, and 11% provided supplementary data, but none provided a supplementary code. See Supplementary Table S1 for additional study characteristics.

### 3.5. Sensor Specifications and Protocols

The sampling rate was reported in 88% of the studies, with the most common rate being 100 (range: 32–2000) Hz. The dynamic range of the inertial sensors was poorly reported and presented in only 32% of the studies. The most common placement of sensors was the back/pelvis/torso (*n* = 41), followed closely by the shank (*n* = 40), foot (*n* = 31), thigh (*n* = 27), and head/neck (*n* = 5). Data were collected most often in-lab (*n* = 47), followed by controlled out-of-lab/clinic (*n* = 28), all with highly variable walk lengths. See Table 2 and Supplementary Table S2 for additional inertial sensor and protocol details.

### 3.6. Gait Outcomes

**Figure 3:**
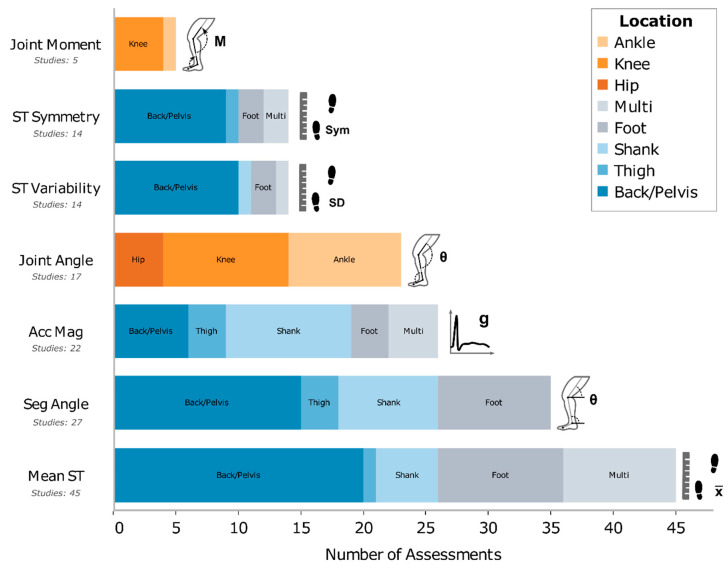
The most reported gait biomechanical outcomes for patients with osteoarthritis, as measured by inertial sensors. Color bands discriminate the sensor placement to obtain spatiotemporal (ST) and accelerometer magnitude measures, whereas the joint angle, segment angle, and joint moment color bands represent the segment or joint of interest. An interactive version of this figure which highlights the referenced studies can be found at: https://public.tableau.com/profile/dylan.kobsar#!/vizhome/WearableOAReview_Story/Fig2_Fig3_Story.

Mean ST parameters were the most common outcome as they were presented in 45 studies. The variability and symmetry of ST parameters were each presented in 14 studies. The second most common outcome was segment orientation or joint angles measured in 33 studies. Linear acceleration magnitudes were the third most common and appeared in 22 studies, with estimates of variability or symmetry components surrounding these outcomes present in 10 studies. Lastly, machine learning analyses were utilized in 12 studies. See Figure 3 for a visual representation of gait outcomes by locations, as well as Supplementary Table S2 for additional details.

## 4. Discussion

Wearable inertial sensors provide an accessible and affordable tool to support our understanding and treatment of OA through gait analyses. We identified 72 studies that utilized wearable inertial sensors to assess OA gait. Overall, these studies measured a wide range of outcomes from mean ST parameters to KAM and more. Similarly, the patient populations, study designs, and sensor protocols varied greatly between the studies. Two-thirds of these studies were published in the past five years (Figure 2) and we predict that 2019 may be an inflection point whereafter remarkable growth will occur in this area for years to come. Our hope is that this scoping review will support this growth by summarizing the current body of literature, with a focus on gait biomechanical outcomes (Section 4.1, Section 4.2, Section 4.3, Section 4.4, Section 4.5, ; Figure 3) and trends for future research (Section 4.6).

### 4.1. Mean Spatiotemporal Parameters

Mean ST parameters were identified in approximately two-thirds of the studies, making them the most common outcome in this scoping review. Not only do ST parameters happen to be some of the most tangible biomechanical outcomes for end-users, but they can be highly effective in comparing disease severities [9,91]. Specifically, a review by Mills et al. (2013 [9]) found that stride time was the strongest and most consistent biomechanical deviation in adults with severe knee OA, as compared to healthy controls. Similarly, numerous studies in this scoping review found that mean ST parameters displayed significant differences between healthy controls and adults with OA [20,21,28,31,35,40,75,80]. However, these studies were generally comparing adults with severe OA or post-total joint arthroplasty (TJA) to healthy controls. Therefore, these findings support the results of Mills et al. (2013 [9]) and highlight the importance of mean ST parameters in more severe OA cohorts.

Given the large number of studies utilizing these mean ST parameters as outcomes, it comes as no surprise that the protocols were highly variable. While a single sensor at the back was the most common placement, numerous studies utilized multiple sensors at a variety of locations. Nevertheless, a recent meta-analysis [15] found that inertial sensors displayed moderate to excellent validity and reliability for mean ST parameters across a range of placements in healthy adult walking. These findings were supported by the current review, as numerous studies found a high level of validity and reliability in these parameters for adults with OA [19,30,37,54,57,67,77]. Only Item-Glatthorn et al. (2012 [47]) cautioned against the use of certain parameters such as double support, walking speed, and step length. However, these results were based on proprietary algorithms sampling bi-axial accelerometers at 32 Hz, well below the recommended rate of 100 Hz [92]. Moreover, the lack of additional sensors (e.g., gyroscope, magnetometer) may have limited the accuracy in detecting toe-off for double support and would have certainly limited the accuracy of spatial measurements such as step length and gait speed. Nevertheless, when using published and appropriately validated algorithms, mean ST parameters obtained from inertial sensors at various locations provide a trustworthy and effective method to assess the gait of adults with OA, especially those in more severe stages of the disease.

### 4.2. Spatiotemporal Parameter Variability

While less commonly assessed than mean ST parameters, the variability of ST parameters represents an important factor to consider in OA gait. Variability outcomes are calculated as the standard deviation or coefficient of variation for a given ST parameter. They are often used as measures of health status, mobility, function, or fall risk, as they are thought to be more sensitive to neurological and/or musculoskeletal changes than mean ST parameters [93,94]. We found that ST parameter variability was examined in 14 studies [21,24,25,26,27,28,35,38,42,63,66,74,79,85] and a variety of applications in OA.

First, ST parameter variability was measured before and after TJA in seven studies [25,26,27,38,42,74,85]. In nearly all instances, ST parameter variability was not the primary focus of the analysis, but rather part of a larger set of outcomes seeking to quantify changes in gait and function with surgery. Fransen et al. (2019 [38]) provides an excellent example of this, as they conducted a factor analysis to summarize a variety of gait measures taken before and after TJA. They found a composite measure of gait quality, dominated by variability outcomes, to be an objective and sensitive measure of functional improvements 1 year following surgery. Alternatively, Hiyama et al. (2018 [42]) was the only study whose primary purpose was to examine changes in ST parameter variability following surgery. However, their purpose was more specifically to examine the immediate changes in stride time variability upon discharge. This was assessed as an indicator of fall risk in the days following total knee arthroplasty (TKA) surgery, rather than a positive outcome of the surgery itself. Therefore, the data were collected only 5 days post surgery and while the authors found knee ROM and gait speed were reduced, gait variability remained unchanged. This finding was contrary to their hypothesis but the lack of regression in this measure even immediately following surgery suggests the potential for further benefits following recovery, similar to Wada et al. (2019 [85]).

Cross-sectional studies also utilized ST parameter variability to compare the gait of asymptomatic controls and adults with OA. While three studies identified differences in these outcomes between healthy and OA cohorts [21,24,28], two studies did not [35,79]. Studies that did not identify a difference in OA and healthy gait tended to examine less severe cohorts, and did so in smaller sample sizes (i.e., <20 OA). Additionally, the walk length used to obtain these measures varied greatly between the studies (20 m [24,28], 60 s [21], 600 s [35,79]), making it difficult to draw any strong conclusions from these findings.

While measures of ST parameter variability may offer a more sensitive assessment of gait health than mean values alone, the lack of consistency in the protocols and processing techniques remains a limiting factor. Lord et al. (2011 [93]) identified this lack of consistency and detailed reporting of protocols for measures of variability nearly 10 years ago and highlighted the need for information such as: walking distance, number of steps, and processing details/rationale for variability outcomes. In general, the reporting of details and protocols may have improved in these studies, but a lack of consistency remains. Furthermore, given that measures of ST parameter variability are based on the fluctuations of individual steps or strides, they are more susceptible to random measurement error [15]. Therefore, future research should seek to evaluate these study protocols in detail to determine the most valid and reliable, as well as practical, protocol and processing techniques for ST parameter variability.

### 4.3. Knee Joint Angles

The knee is the most common joint for OA diagnosis [95] and as such, the knee continues to dominate the literature around OA gait kinematics. A total of 10 studies measured knee angles during gait in adults with OA [20,29,39,40,46,58,64,78,81,90], as compared to eight measuring ankle angles [31,32,33,39,46,64,70,71] and only four measuring hip angles [39,46,80,90]. Not surprisingly, examining differences in sagittal plane knee angles between OA and the control was the most common application [20,39,40,58,64,78]. Unfortunately, the quality of these studies was highly variable (e.g., 36–75%) and only two recruited more than 20 adults with OA [58,64]. One such example, Rahman et al. (2015 [64]), cross-sectionally examined the gait of 74 individuals with knee OA at different stages of surgery and recovery as compared to healthy controls. They found an inertial sensor-derived sagittal knee joint range of motion was reduced in the OA populations as compared to healthy controls. Furthermore, patients one-year post surgery had an improved range of motion compared to those pre-surgery, but they remained below their healthy counterparts. Alternatively, Calliess et al. (2014 [29]) was the only study to directly examine sagittal knee joint angles before and after surgery in a prospective manner. Unfortunately, this was an initial evaluation on only six patients and the authors were unable to identify a significant change in peak knee flexion with surgery.

Given the limited number of studies examining knee joint angles with wearable sensors in OA, there is lack of consensus on the system and processing. The 10 studies identified in this area used a variety of sensor systems, some involving proprietary algorithms in clinically-focused sensors [39,46,58,64,90] and others involving raw inertial sensor data from research-focused sensors [20,29,40,78,81]. While the exact algorithms to process these data vary greatly between studies, the procedures generally involved some type of attitude correction and/or sensor alignment, segment orientation estimation, knee joint angle calculation, and discrete parameter extraction (i.e., peak angle, range of motion, etc.). Interestingly, no study reported using machine learning algorithms, Kalman filters, or complementary filters in the estimation of the sagittal knee joint angle, however, most utilized a short-range drift correction to account for the errors in angular velocity estimation [29,40,78]. Although a variety of methods existed, studies examining the validity of these outcomes [20,40,90] came to similar conclusions as in previous work. Specifically, sagittal knee joint angles display excellent agreement with gold-standard motion capture systems [15,96,97]. While there is still a need for greater consistency in the systems and methodology, the current body of literature supports the use of these outcomes in larger prospective studies.

### 4.4. Joint Moments

Estimating the loading environment of an osteoarthritic joint has been a primary focus of gait biomechanics. Specifically, in knee OA, KAM remains a strong predictor of structural progression [10,11,12] and a key outcome examined for gait retraining [98,99] and surgical interventions [100]. Previous research has demonstrated the ability of inertial sensors to estimate joint moments, but this has most often occurred in healthy populations [101,102,103]. Alternatively, we identified three studies which examined KAM [41,83,86], as well as one examining joint contact forces [36] and one examining ankle joint moments [70]. The first proof of principle study was conducted by van den Noort et al. (2013 [83]), which used eight inertial sensors on the lower limbs, in combination with instrumented force shoes, to estimate KAM. While successful at the group level, significant errors remained on an individual level which related to the estimation of joint centres, sensory orientation/alignment, and inaccuracies in force measurements. More recently, Wang et al. (2020 [86]) and He and Liu (2019 [41]) evaluated the use of patient-specific inertial sensor systems which involved only one sensor per ankle to estimate KAM in real-time. The inertial sensor applications required a short calibration using data from a conventional system before a patient-specific model could be created to track real-time changes in KAM using only inertial sensors. While these are interesting interim approaches to track patient-specific changes in KAM with inertial sensors, they are not independent systems. Overall, this remains an exciting new area of study that requires further advancements before inertial sensor-based joint moment outcomes can be effectively measured in a clinically viable manner.

### 4.5. Acceleration Magnitudes

In addition to the more conventional biomechanical outcomes already discussed, inertial sensors offer the ability to measure several unique impact-related outcomes. Specifically, impact accelerations were found to be among the most common biomechanical outcomes, following ST parameters and joint/segment kinematics. Segmental accelerations can be examined using a variety of methods, such as the overall amount of multi-axis accelerations (e.g., root mean square [23,30,56,63,72,82] or mean [30,88]), impacts peaks [21,25,28,34,45,48,56,59,60,81,82,87], or waveform analyses [50,51,52]. These outcomes have been shown to be reliable in healthy adults [15] and adults with knee OA [52,56,82], and can be measured at a variety of placements including the lower back [21,23,25,28,50,51,52,63,72], thigh [50,51,52,81,82], shank [34,45,48,50,51,52,56,59,60,81,82,87], and foot [23,30]. While for most of the studies we identified examined cross-sectional differences between OA and healthy gait, these outcomes may provide important and objective information that can supplement the clinical outcomes of TKA [25,34,51,72,88].

Lastly, the regularity and symmetry of acceleration patterns have been increasingly examined to measure dynamic stability in OA gait. These measures utilize either an autocorrelation procedure to examine waveform similarity between steps or strides [104] or a frequency analysis to examine asymmetrical harmonics [105]. The popularity of such metrics continues to grow given their ease of use and sensitivity to changes in gait patterns following interventions [38,43,65]. Nevertheless, while these outcomes have demonstrated moderate to good reliability in healthy adults, we did not identify any assessments of reliability in adults with OA. Therefore, there is a need to standardize the testing protocol and processing techniques, as well as better understand the underlying biomechanical relevance, before wearable sensor regularity and symmetry outcomes can be used effectively in a clinical setting.

### 4.6. Trends for Future Research

Our scoping review has demonstrated the immense growth and success of inertial sensors in assessing the gait of adults with OA. As the burden of this disease continues to grow [3], so must our efforts to offset it. Wearable inertial sensors offer a unique opportunity to support this endeavor but will require advancements in several key areas.

Longitudinal designs—There is a breadth of cross-sectional research demonstrating the validity, reliability, and sensitivity of many inertial sensor-derived gait outcomes in OA. While the more recent integration of inertial sensors in surgical intervention studies is promising, the majority of these studies have focused only on mean ST parameters as outcomes. Given the body of literature presented in this review, we are confident that inertial sensors can effectively examine additional biomechanical outcomes (e.g., sagittal knee joint angle, impact accelerations, etc.) in these studies. Nevertheless, an even more glaring gap in the literature is a lack of studies utilizing inertial sensors to examine gait in relation to disease progression. Consequently, to truly strengthen the quality of evidence surrounding inertial sensor biomechanical outcomes in OA, future research must utilize more longitudinal study designs that can highlight change and progression over time.

Patient-specific applications—In addition to tracking group-level changes in gait, inertial sensors offer an exciting opportunity to develop personalized treatment plans through monitoring patient-specific changes [106]. For instance, both Kwasnicki et al. (2015 [53]) and Kobsar et al. (2018 [50]) demonstrated the utility of tracking gait outcomes as they relate to a patient’s response to a surgical or exercise intervention, respectively. Tracking patient-specific changes in gait, assessed by a variety of outcomes collected over multiple days, can provide sensitive, objective, and clinically relevant outcomes for OA. Nevertheless, there is a need to better understand the normal day-to-day fluctuations in natural, free-living gait patterns which can support our understanding of what clinically meaningful levels of change are.

Free-living assessments—Wearable sensors are already expanding our capability to assess OA gait, as 63% of studies involved some out-of-lab component. However, in accordance with a previous review on wearable sensors in out-of-lab settings [92], these collections still rarely support natural or free-living walking. In other words, even those gait assessments done at home or in a clinical setting still involve predetermined walking paths or walk lengths that may poorly represent a patient’s normal gait pattern, and as such misrepresent the typical loading environment experienced by their joints. Unfortunately, free-living assessments require the integration of robust activity classification and event detection algorithms, which are often studied independently but rarely implemented together in a single computational pipeline or system [13,107]. The development of robust pipelines such as these could expand the use of inertial sensors to track changes in free-living gait patterns related to acute flares, interventions, and even progression.

Push for “Code Reuse”. Both open access publishing and open source software repositories present important models which can support the growth, replication, consistency, and inclusivity across clinical research [108,109]. We found that 44% of the studies identified in this review were open access, which is well above the 28% average [108]. Unfortunately, there were no studies that provided any open source software to accompany their articles. Publishing software to promote “Code Reuse” enables cost-effective replication and advancement software for clinical research [108]. While limited examples do exist in the broad field of wearable technology [107,110], a concerted effort to make the code more accessible to other researchers will be one of the most important trends in advancing wearable inertial sensor applications to gait biomechanics.

## 5. Conclusions

Wearable inertial sensor research is growing at an exceptional rate and expanding how biomechanical analyses can be used for patients with lower limb OA. While mean ST parameters remain the most assessed outcomes in OA, recent work has highlighted the ability of inertial sensors to measure more advanced outcomes such as knee joint angles, KAM, and impact accelerations, as well as a variability and symmetry measures. Nevertheless, there remains a need for more longitudinal study designs, patient-specific models, free-living assessments, and a push for “Code Reuse” to maximize the unique capabilities of these devices and ultimately improve how we diagnose and treat this debilitating disease.

## Figures and Tables

**Table 1:** Quality assessment scoring of all 72 included studies.

Study Information			Study Ques	Study Design					Measurement		Analyses			Rec	Score		
Ref	Author	Year	Q1	Q2	Q3	Q4	Q5	Q6	Q7	Q8	Q9	Q10	Q11	Q12	/24	%	Rating
[19]	Aminian	1999	2	1	1	1	0	2	2	1	1	1	1	1	14	58	Low
[20]	Aminian	2004	1	1	1	0	0	NA	2	0	1	0	1	1	8	36.4 *	V. Low
[21]	Andrade	2017	2	2	1	1	0	NA	1	1	1	2	1	2	14	63.6 *	Low
[22]	Auvinet	1999	1	1	0	0	0	NA	1	2	1	1	2	1	10	45.5 *	V. Low
[23]	Barrois	2016	2	2	1	0	0	NA	2	2	1	1	2	2	15	68.2 *	Low
[24]	Bolink	2015	2	1	2	0	0	NA	2	1	2	1	1	2	14	63.6 *	Low
[25]	Bolink	2015	2	1	2	1	0	2	2	1	1	2	2	2	18	75	Mod
[26]	Bolink	2016	2	1	2	2	0	1	2	1	1	2	1	1	16	66.7	Low
[27]	Bolink	2019	2	1	1	1	0	2	1	1	2	1	2	1	15	62.5	Low
[28]	Bolink	2012	2	0	2	0	0	NA	2	1	1	1	1	2	12	54.5 *	Low
[29]	Calliess	2014	2	2	2	1	0	2	2	2	1	1	2	1	18	75	Mod
[30]	Chen	2016	2	1	1	0	0	NA	1	2	2	1	1	1	12	54.5 *	Low
[31]	Chopra	2019	2	1	2	1	0	NA	1	1	1	1	1	1	12	54.5 *	Low
[32]	Chopra	2017	2	1	1	1	0	NA	2	2	2	1	1	2	15	68.2 *	Low
[33]	Chopra	2014	1	1	2	1	0	NA	1	2	2	1	2	1	14	63.6 *	Low
[34]	Christiansen	2015	2	2	2	2	0	0	1	2	1	1	2	1	16	66.7	Low
[35]	Clermont	2016	2	2	2	1	0	NA	2	1	1	1	2	2	16	72.7 *	Mod
[36]	De Brabandere	2020	2	1	2	1	0	NA	1	1	2	1	1	1	13	59.1 *	Low
[37]	De Vroey	2018	2	2	2	1	0	NA	2	2	1	2	2	2	18	75.0 *	Mod
[38]	Fransen	2019	2	2	1	1	2	1	2	2	2	1	2	1	19	79.2	Mod
[39]	Grip	2019	2	2	2	1	0	NA	2	2	2	1	1	1	16	72.7 *	Mod
[40]	Hafer	2020	1	1	2	1	0	NA	2	1	1	1	1	1	12	54.5 *	Low
[41]	He	2019	2	1	2	1	0	NA	1	1	2	1	1	2	14	63.6 *	Low
[42]	Hiyama	2015	1	2	1	2	0	1	2	1	2	1	2	2	17	70.8	Mod
[43]	Hiyama	2020	1	2	2	1	0	2	2	2	2	2	2	1	19	79.2	Mod
[44]	Iijima	2019	2	2	2	1	2	NA	1	1	2	2	1	1	17	77.3 *	Mod
[45]	Ishii	2020	2	2	2	1	0	NA	2	1	2	2	2	1	17	77.3 *	Mod
[46]	Ismailidis	2020	1	2	1	0	2	NA	1	1	1	2	1	1	13	59.1 *	Low
[47]	Item-Glatthorn	2012	1	1	1	2	0	NA	1	1	1	2	1	1	12	54.5 *	Low
[48]	Khan	2013	1	1	2	1	0	NA	2	2	1	1	0	1	12	50	Low
[49]	Kluge	2018	2	1	2	1	0	1	1	1	1	2	2	2	16	66.7	Low
[50]	Kobsar	2018	2	2	2	1	0	NA	2	2	2	1	2	1	17	77.3 *	Mod
[51]	Kobsar	2017	2	2	2	1	0	2	1	1	2	2	2	2	19	79.2	Mod
[52]	Kobsar	2016	2	2	2	1	0	1	2	2	2	2	1	1	18	75	Mod
[53]	Kwasnicki	2015	1	1	1	2	0	2	2	2	2	1	1	1	16	66.7	Low
[54]	L’Hermette	2008	1	1	1	1	0	NA	2	2	1	1	1	1	12	54.5 *	Low
[55]	Liikavainio	2010	1	2	2	1	0	NA	1	1	2	1	2	2	15	68.2 *	Low
[56]	Lyytinen	2016	2	1	2	1	0	NA	2	2	2	1	2	2	17	77.3 *	Mod
[57]	Mariani	2013	1	1	0	1	0	NA	2	2	2	1	0	2	12	54.5 *	Low
[58]	McCarthy	2013	2	2	2	1	0	NA	2	2	1	1	2	1	16	72.7 *	Mod
[59]	Na	2019	2	2	2	1	0	NA	2	2	2	2	2	1	18	81.8 *	Mod
[60]	Na	2020	2	1	1	2	0	NA	2	1	1	0	2	1	13	59.1 *	Low
[61]	Nelms	2020	2	2	2	1	0	1	1	1	1	2	1	1	15	62.5	Low
[62]	Odonkor	2020	2	2	2	1	0	NA	2	2	1	2	0	1	15	68.2 *	Low
[63]	Oka	2019	1	2	1	1	0	NA	2	2	2	1	2	1	15	68.2 *	Low
[64]	Rahman	2015	2	2	2	1	0	NA	1	1	1	1	1	2	14	63.6 *	Low
[65]	Rapp	2015	2	1	2	2	0	1	2	2	1	1	2	2	18	75	Mod
[66]	Reh	2019	2	1	1	1	0	1	1	1	1	1	2	2	14	58.3	Low
[67]	Reininga	2011	2	1	2	1	0	NA	2	1	1	2	0	2	14	63.6 *	Low
[68]	Reininga	2012	2	1	2	1	0	NA	2	2	2	2	1	2	17	77.3 *	Mod
[69]	Rouhani	2012	2	1	2	1	1	NA	2	1	2	1	2	2	17	77.3 *	Mod
[70]	Rouhani	2014	1	1	2	1	0	NA	1	1	2	1	2	1	13	59.1 *	Low
[71]	Rouhani	2012	1	1	0	1	0	NA	2	1	1	1	0	1	9	40.9 *	V. Low
[72]	Saida	2020	2	1	2	1	0	NA	1	2	1	2	2	2	18	75.0 *	Mod
[73]	Samani	2020	2	1	2	1	0	NA	2	2	1	1	1	1	14	63.6 *	Low
[74]	Senden	2011	2	1	2	2	0	1	2	2	2	1	0	1	16	66.7	Low
[75]	Staab	2014	1	1	1	1	0	NA	2	1	1	1	0	1	10	45.5 *	V. Low
[76]	Suh	2019	2	2	1	2	1	NA	0	1	2	1	2	1	15	68.2 *	Low
[77]	Sun	2017	1	0	2	1	0	NA	2	2	1	1	1	0	11	50.0 *	Low
[78]	Tadano	2016	1	1	1	1	0	NA	2	1	2	1	1	1	12	54.5 *	Low
[79]	Tanimoto	2017	1	1	2	1	0	NA	2	2	1	1	2	1	14	63.6 *	Low
[80]	Teufl	2019	2	1	1	1	0	NA	2	1	2	2	2	2	16	72.7 *	Mod
[81]	Turcot	2008	2	2	1	1	0	NA	2	1	2	1	1	1	14	63.6 *	Low
[82]	Turcot	2008	2	1	2	2	0	NA	1	2	2	2	0	1	15	68.2 *	Low
[83]	van den Noort	2013	2	1	2	1	0	NA	2	2	1	1	2	1	15	68.2 *	Low
[84]	van Hemert	2009	2	1	2	0	0	NA	1	1	2	1	2	2	14	58.3 *	Low
[85]	Wada	2019	1	2	2	2	0	2	2	1	2	2	2	2	20	83.3	Mod
[86]	Wang	2020	2	2	0	1	2	NA	2	2	2	1	2	1	17	77.3 *	Mod
[87]	Youn	2018	1	1	1	1	0	NA	2	2	2	1	1	1	13	59.1 *	Low
[88]	Zhang	2016	2	1	1	1	0	2	1	2	2	1	1	1	15	62.5	Low
[89]	Zijlstar	2008	0	1	1	1	0	NA	2	1	2	1	0	1	10	41.7 *	V. Low
[90]	Zugner	2019	2	1	2	1	0	NA	2	2	1	2	2	1	16	72.7 *	Mod

* percentage calculated out of 22 as studies did not involve repeated measures.

**Table 2:** Summary of the samples and protocols for all 72 included studies. Additional information can be found in Supplementary Tables S1 and S2.

Study			Sample				Sensors				Protocol	
Ref	Author	Year	n	%F	Age	BMI	Range (±g)	Frequency (Hz)	# Sensors	Placement	Setting	Walk Length
[19]	Aminian	1999	12H	33	64.6 (8.6)	27.9 (2)	±5	60	2(b)	T	IL	70 m
[20]	Aminian	2004	19H		63.8 (6.9)	26.5		200	4(b)	TS	IL	10 m
[21]	Andrade	2017	24H		65 (8.5)		±16	50	2(b)	B	IL	60 s
[22]	Auvinet	1999	42HK	47	67.4 (7.3)	27.1		50	1(u)	B	OL	40 m
[23]	Barrois	2016	48HK		70.5 (12.2)	27.5 (5.6)	±16	100	4(b)	HBF	OL	20 m
[24]	Bolink	2015	40HK	53	64.7 (8.9)	28.7 (6.1)		100	1(u)	B	OL	20 m
[25]	Bolink	2015	20K	65	67.4 (7.7)			100	1(u)	B	OL	20 m
[26]	Bolink	2016	36H	50	63.9 (9.8)	26.3 (3.5)	±5	100	1(u)	B	OL	20 m
[27]	Bolink	2019	77H	52	65 (11)	27 (5)	±5	100	1(u)	B	IL	20 m
[28]	Bolink	2012	20H	65	67.4 (7.7)		±5	100	1(u)	B	OL	20 m
[29]	Calliess	2014	6K	50	60.2	26.1			3(u)	BTS	IL	100 m
[30]	Chen	2016	14H	79	57.2	25		50	2(b)	F	IL	15 m
[31]	Chopra	2019	10A		65.8 (8.9)	27.6 (3)		200	5	SF	OL	50 m
[32]	Chopra	2017	24A					200	6(b)	SF	OL	50 m
[33]	Chopra	2014	24A	46	64.6 (9)	27.7 (4.7)			5	SF	OL	50 m
[34]	Christiansen	2015	24K	54	65.2 (9.2)	28.9	± 10	1000	2(b)	SF	OL	6 m
[35]	Clermont	2016	15K		64.6 (6.8)	30.6 (4)		100	1(u)	B	OL	600 s
[36]	De Brabandere	2020	20H					50	1(u)	T	IL	
[37]	De Vroey	2018	16K		64.1 (7.5)	32.2		100	2(u)	SF	OL	6 m
[38]	Fransen	2019	65K	54	65	30	±6	100	1(u)	B	OL	50 m
[39]	Grip	2019	15H	0	51.8 (9)	27.4 (3.2)	±10	128	5(b)	BTS	OL	9 m
[40]	Hafer	2020	9K	44	69.2 (4.5)	26.2	±16	128	4(u)	BTSF	IL	10 m
[41]	He	2019	6K	100	60.8 (1.1)	27.4 (0.6)		200	1(u)	F	IL	10 m
[42]	Hiyama	2015	43K	81	72 (6.6)	25.9 (3.3)		500	1(u)	F	IL	10 m
[43]	Hiyama	2020	27K	85	71 (6)	25.9 (3)		500	2(u)	BF	IL	10 m
[44]	Iijima	2019	131K	72	74.2 (5.8)	21.7 (2.5)		200	1(u)	B	IL	20 m
[45]	Ishii	2020	44K	50	68.9 (9.3)	25.1 (3.1)		100	2(u)	SF	IL	20 m
[46]	Ismailidis	2020	23K	48	66.1 (8.9)	28.1 (3.8)			7(b)	BTSF	IL	20 m
[47]	Item-Glatthorn	2012	26H	0	54 (9)	27.1		32	5	BTSF	IL	
[48]	Khan	2013	38K	42			± 2	100	1(u)	SF	IL	
[49]	Kluge	2018	24K	67	64 (11)	31.3 (6.8)	±8	102.4	2(b)	F	IL	40 m
[50]	Kobsar	2018	8K	50	58 (5)	25.3 (4.8)	±16	100	4(u)	BTSF	IL	150 s
[51]	Kobsar	2017	39K		59 (8)	26.6 (3.8)	±16	100	4(u)	BTSF	IL	60 s
[52]	Kobsar	2016	10K	40	57 (8)	26 (4.5)	±16	100	4(u)	BTSF	IL	60 s
[53]	Kwasnicki	2015	14K	57	69.3 (4.6)	29.2 (2.8)	±3	50	1(u)	H	OL	
[54]	L’Hermette	2008	5H	0	72.3 (9.5)			100	1(u)	B	IL	50 m
[55]	Liikavainio	2010	54K	0	59 (5.3)	29.7 (4.7)	±10	1000	3	TS	IL/OL	10 m
[56]	Lyytinen	2016	9K	0	62.7 (5.1)	27 (4.2)		1000	1(u)	S	OL	15 m
[57]	Mariani	2013	34A	24	63.8 (17)	28.1		200	1(u)	F	OL	50 m
[58]	Mccarthy	2013	23K	61	65.1 (7.7)	28.7 (3.7)		102.4	4(b)	TS	OL	20 m
[59]	Na	2019	26K	62	66 (6.1)	30.6 (5.6)		100	5(b)	BTS	IL	50 m
[60]	Na	2020	26K	62	65.9 (6.1)	30.5 (5.6)			5(b)	BTS	IL	10 m
[61]	Nelms	2020	69H	47	61.2 (8.1)	26.8 (4.9)			1(u)	B	IL	14 m
[62]	Odonkor	2020	10K	60	63.9 (8.1)	33.2 (8.4)		102.4	2(b)	F	IL	6 m
[63]	Oka	2019	41K	100	72.3 (7.1)	26 (3.9)		200	2(b)	HB	IL	20 m
[64]	Rahman	2015	45K	57	66.9 (10.7)	29.9 (4.7)			5(b)	TS	OL	20 m
[65]	Rapp	2015	29H	48	67.8 (6.3)	24.9 (4.9)		100	1(u)	B	IL	20.3 m
[66]	Reh	2019	20H	20	63 (8.6)	27.5		60	7(u)	BTSF	IL	20 min
[67]	Reininga	2011	15H		61 (9)	25.6		100	2(b)	HB	IL/OL	33 m
[68]	Reininga	2012	60H	75	59.7 (8.7)	26.6		100	2(u)	HB	OL	25 m
[69]	Rouhani	2012	35A	26	63.5 (18.6)	28.1		200	4(u)	SF	OL	50 m
[70]	Rouhani	2014	12A	33	58 (13)	28.4		200	4(u)	SF	OL	100 m
[71]	Rouhani	2012	15A	26	53.3	28		200	3	SF	OL	100 m
[72]	Saida	2020	18K	67	72 (9)	25.9 (2)		100	3(u)	BS	IL	10 m
[73]	Samani	2020	19K	47	66.2 (5.2)	28.1 (2.7)		2000	8(u)	TS	IL	40 m
[74]	Senden	2011	24K	54	70 (8)	27.3 (4)		100	1(u)	B	OL	20 m
[75]	Staab	2014	12K	17	44.4 (7.6)	26.9 (3.2)	±2	1000	3(b)	BS	IL	500 m
[76]	Suh	2019	195K	84	72.6 (6.1)	26 (3.1)			1(u)	B	OL	8 m
[77]	Sun	2017	23K		69.9 (6.6)	26.6 (3)		32	7(b)	BTSF	IL/OL	16 m
[78]	Tadano	2016	10K		68.7 (4.1)	23.5 (2.5)	±4	100	7(b)	BTSF	IL	7 m
[79]	Tanimoto	2017	12K	83	73	23.4 (2.5)	±2	100	1(u)	S	IL	600 s
[80]	Teufl	2019	20H	65	56.9 (8.2)	27.4		60	5(b)	BTSF	IL	7 m/600 s
[81]	Turcot	2008	9K	67	63.4 (4.6)	32.2	±5	120	4(b)	TS	IL	
[82]	Turcot	2008	25K	76	63.9 (7.6)	31.6	± 5	120	4(b)	TS	IL	
[83]	van den Noort	2013	14K	79	61 (9.2)	30.4		50	8(b)	TSF	IL	10 m
[84]	van Hemert	2009	53K		71.9 (8.3)	28.3 (3.9)			6(u)	BTS	IL	20 m
[85]	Wada	2019	23H	100	61 (7.1)	23 (3.2)		500	2(u)	BF	IL	10 m
[86]	Wang	2020	78K	57	59.7 (7.1)	23 (3.8)	±4	100	2(b)	S	IL	20 m
[87]	Youn	2018	18K	50	66.5 (7.7)	29.5 (4.9)		200	2(b)	S	IL	6 m
[88]	Zhang	2016	12K	58	65.3 (8)	26.6 (3.5)			7(b)	BTF	IL	40 m
[89]	Zijlstra	2008	4K	50				100	2(u)	B	IL	30 m
[90]	Zügner	2019	49H	49	73	28.7		102.4	6(b)	BTS	IL	

Abbreviations—(i) sample: H = hip, K = knee, A = ankle, %F = percent female; (ii) placement: H = head, B = back/pelvis/torso, T = thigh, S = shank, F = foot; (iii) setting: IL = in laboratory, OL = outside laboratory. “# Sensors” refers to “Number of Sensors”.

## Supplementary Materials

The following are available online at https://www.mdpi.com/1424-8220/20/24/7143/s1, Table S1: Additional details on study designs and samples for all 72 included studies, Table S2: Additional details on inertial sensors, protocols, and biomechanical variables for all 72 included studies.

## Abbreviations

The following abbreviations are used in the manuscript:

OA	Osteoarthritis
KAM	Knee Adduction Moment
ST	Spatiotemporal
PRISMA	Preferred Reporting Items for Systematic Reviews and Meta-Analyses
BMI	Body Mass Index
TJA	Total Joint Arthroplasty
TKA	Total Knee Arthroplasty

## Appendix A

Complete inclusion and exclusion criteria.

Inclusion Criteria:

(i) Studies include the use of a wearable inertial sensor (e.g., accelerometers, gyroscopes, and/or magnetometers) for gait analyses;
(ii) Studies involve unassisted, walking gait analyses on patients with lower limb osteoarthritis (e.g., hip, knee, or ankle);
(iii) Studies are published in English.

Exclusion Criteria:

(i) Conference abstracts;
(ii) Systematic reviews;
(iii) Published study protocols;
(iv) Osteoarthritis was not diagnosed either radiographically or clinically by a health professional;
(v) Population-based cohorts with a mixed sample of clinical conditions (i.e., not clearly separating osteoarthritis patients from other conditions);
(vi) Daily step count, physical activity, or energy expenditure assessments only;
(vii) Activity classification or pedestrian navigation only;
(viii) Non-human subjects.

## Appendix B

Complete search strategy.

Search strategy individually optimized for each database based on the three broad topics of inertial sensors, gait, and osteoarthritis joined using the AND search command/function.

**Medline/Embase:**

Inertial Sensors: (wearable sensor* or wearable technology or motion sensor* or inertial sensor* or inertial motion capture or inertial measurement unit* or body sensor network* or body worn sensor* or sensor fusion or smartphone* or IMU or MEMS*or acceleromet* or gyroscop* or magnetomet*).mp. or exp accelerometer/

Gait: ((speed* or time* or length* or width or cadence* or spatiotemporal or analys* or kinematic* or kinetic* or biomechanic* or angle* or acceleration*) adj5 (step or stride or gait* or walk* or segment* or joint* or hip* or knee* or ankle* or torso* or center of mass or centre of mass or center of gravity or centre of gravity)).mp. or exp biomechanics/

Osteoarthritis: (osteoarthritis or degenerative arthritis or degenerative joint disease).mp. or hip osteoarthritis/ or osteoarthritis/ or knee osteoarthritis/

**Cinahl/SPORTDiscus:**

Inertial Sensors: (wearable sensor* or wearable technology or motion sensor* or inertial sensor* or inertial motion capture or inertial measurement unit* or body sensor network* or body worn sensor* or sensor fusion or smartphone* or IMU or MEMS*or acceleromet* or gyroscop* or magnetomet*)

Gait: ((speed* or time* or length* or width or cadence* or spatiotemporal or analys* or kinematic* or kinetic* or biomechanic* or angle* or acceleration*) N5 (step or stride or gait* or walk* or ambulat* or segment* or joint* or hip* or knee* or ankle* or torso* or “cent* of mass” or “cent* of gravity))

Osteoarthritis: (osteoarthritis or degenerative arthritis or degenerative joint disease)

**Web of Science core collection:**

Inertial Sensors: TS = (wearable sensor* or wearable technology or motion sensor* or inertial sensor* or inertial motion capture or inertial measurement unit* or body sensor network* or body worn sensor* or sensor fusion or smartphone* or IMU or MEMS*or acceleromet* or gyroscop* or magnetomet*)

Gait: TS = ((speed or step time* or stride time* or step length* or stride length* or step width* or spatiotemporal or kinematic* or kinetic* or biomechanic* or analys* or (joint near/5 angle*) or (segment near/5 angle*) or (hip near/5 angle*) or (knee near/5 angle*) or (ankle near/5 angle*) or (foot near/5 angle*) or (acceleration* near/5 segment*) or (acceleration* near/5 torso*) or (acceleration* near/5 {centre of mass}) or (acceleration* near/5 {center of mass})) and (gait or walk* or ambulat*))

Osteoarthritis: TS = (osteoarthritis or degenerative arthritis or degenerative joint disease)

**Engineering Village:**

Inertial Sensors: (({wearable sensor*} or {wearable technology} or {motion sensor*} or {inertial sensor*} or {inertial motion capture} or {inertial measurement unit*} or {body sensor network*} or {body worn sensor*} or {sensor fusion} or {smartphone*} or IMU or MEMS*or acceleromet* or gyroscop* or magnetomet*) WN KY)

Gait: ((gait or walk* or ambulat* or speed or step time* or stride time* or step length* or stride length* or step width* or spatiotemporal or kinematic* or kinetic* or biomechanic* or (joint near/5 angle*) or (segment near/5 angle*) or (hip near/5 angle*) or (knee near/5 angle*) or (ankle near/5 angle*) or (foot near/5 angle*) or (acceleration* near/5 segment*) or (acceleration* near/5 torso*) or (acceleration* near/5 {centre of mass}) or (acceleration* near/5 {center of mass})) WN KY)

Osteoarthritis: (osteoarthritis or degenerative arthritis or degenerative joint disease) WN KY

*Note: The variant term “spatio-temporal” (vs. “spatiotemporal”) was not included in the search strategy, as it did not result in any additional studies identified in our complete search strategy. Nevertheless, this may not be the case in other searches/topics, and as such it may still be necessary to include spatio-temporal” in other search strategies.*

## Appendix C

Critical appraisal of study design.

**Interpretation Guide**

To decide which score to provide for each item on a quality checklist, read the following descriptors. Pick the descriptor that sounds most like the study being evaluated with respect to a given item. If there is no documentation of an action, treat it as not done.

Adapted from: Law, MacDermid. Evidence-based rehabilitation: A guide to practice, 2nd edition; Slack Inc.: Thorofare, NJ, USA, 2008.

**Table:**

Question	Score	Descriptors
Study Question		
1 Background and Research Question	2	The authors: 1. Performed a thorough literature review, indicating what is currently known about the area from previous research studies; 2. Presented a critical and unbiased view of the current state of knowledge; 3. Indicated how the current research question or objective evolves from a gap in the current knowledge base.
	1	All of the above criteria were not fulfilled (little reference to previous research and present gaps in knowledge), but a clear rationale was provided for the research question or objective.
	0	A foundation for the current research question or objective was not clear, and the rationale was not founded on previous literature.
Study Design		
2 Subjects	2	Appropriate characteristics of the participants are described in the inclusion/exclusion criteria and reported in the data (i.e., they clearly describe the characteristics of the sample they are looking to recruit).
	1	Appropriate characteristics of the participants are poorly described in the inclusion/exclusion criteria but reported in the data (i.e., they do not clearly describe the characteristics of the sample they are looking to recruit, but they do outline important characteristics after the fact—age, height, weight, BMI, severity, additional conditions).
	0	Appropriate characteristics of the participants are poorly described in the inclusion/exclusion criteria AND poorly reported in the data.
3 Objective/ Hypothesis	2	Authors clearly identified main objectives/hypotheses of the study.
	1	Authors broadly identified main objectives/hypotheses of the study.
	0	Authors did not identify a main objective of the study.
4 Scope/ Design	2	The overall design of the study is clearly described and appropriately linked to addressing the objective/research question.
	1	The overall design of the study is identifiable and adequately addresses the objective/research question.
	0	The design of the study is unclear and/or poorly addresses the purpose of the study.
5 Sample	2	Authors performed a sample size calculation and obtained their recruitment targets.
	1	The authors provided a rationale for the number of subjects included in the study but did not present specific sample size calculations. Alternatively, sample is greater than 100, but has no justification for sample size.
	0	Size of the sample was not rationalized or is clearly underpowered.
6 Retention (if Applicable)	2	Ninety percent or more of the patients enrolled for study were re-evaluated.
	1	More than 70% of the patients eligible for study were re-evaluated or not directly stated (e.g., sampling from a database).
	0	Less than 70% of the patients eligible for study were re-evaluated.
Measurements		
7 Sensor	2	The authors provided detailed information that outlines the measurement device (i.e., inertial sensor) and specific procedures for data collections. This information needs to consist of: (i) name, manufacturer of sensor, and sampling rate, (ii) specifics on placement, and (iii) sensor calibration procedures.
	1	Device is referenced with moderate description/information (2 complete categories out of 3).
	0	Minimal description of device is provided and without appropriate references (0 or 1 categories out of 3).
8 Protocol	2	The authors provided detailed information on the exact protocol of the study which would allow for excellent replication of the study.
	1	The authors provided adequate information on the protocol of the study that would allow for a replication of the study but may miss some specific details.
	0	The authors poorly describe the exact protocol of the study and it would be difficult to replicate the study.
Analyses		
9 Organization	2	Authors clearly defined which analyses were conducted for each of the stated specific research questions/hypotheses of the study. Data were clearly organized and presented for each objective/hypothesis in the results.
	1	Data were presented for each specific objective/hypothesis, but authors did not clearly link these analyses to the defined objective/hypothesis.
	0	Data were not presented for each objective/hypothesis outlined in the purposes or methods.
10 Statistical Analyses	2	Statistical tests used to assess outcomes were clearly described and appropriate (e.g., more than just means (SDs) and significance levels; confidence intervals (CIs) and effect sizes reported).
	1	Statistical tests used to assess outcomes were adequately describe and appear appropriate (e.g., only present means (SDs) and significance levels).
	0	Statistical tests to assess outcomes were poorly described or not appropriate (e.g., missing important information such as means (SDs) or significance levels)
11 Confounding Effects	2	Authors clearly discussed and effectively addressed/acknowledged most confounding effects to the outcomes.
	1	Some potential confounding effects were briefly discussed or minimized.
	0	Confounding effects appear to be prevalent in the results and not discussed or acknowledged.
Recommendations		
12 Conclusion/ Recommendations	2	Authors made specific conclusions (based on results) and clinical recommendations that were clearly related to the specific objectives/hypotheses stated at the beginning of the study and supported by the data presented.
	1	Authors made conclusions and clinical recommendations that were general, but basically supported by the study data, OR authors made conclusions and clinical recommendations for only some of the study objective/hypotheses.
	0	Authors made vague conclusions without any clinical recommendations, and conclusions OR recommendations contradicted the actual data presented.
