# Supplementary material for: Wearable Inertial Sensors for Gait Analysis in Adults with Osteoarthritis—A Scoping Review
Source: Sensors (Basel). 2020 Dec 13;20(24):7143. doi: 10.3390/s20247143 (PMC7763184; doi:10.3390/s20247143)
Supplement: Supplementary file 1 [file sensors-20-07143-s001.zip › Supplementary Materials/Supplementary_Table1.pdf]

Supplementary Table 1: Additional details on study designs and samples for all 72 included studies.

Study Information			Study Design			Sample								Publication		
Ref	Author	Year	Longitudinal	Cross-sectional	Validation/ Reliability	n-OA	% Female	Age	BMI	Mild-Mod.	End-stage	Post-TJA	Control group	Open access	Supp. data	Supp. code
19	Aminian	1999	S			12H	33	64.6 (8.6)	27.9 (2)			x	x			
20	Aminian	2004		OC/OS	V	19H		63.8 (6.9)	26.5		x		x			
21	Andrade	2017		OC		24H		65.0 (8.5)				x	x			
22	Auvinet	1999		OC	V	42HK	47	67.4 (7.3)	27.1				x	x		
23	Barrois	2016		OS		48HK		70.5 (12.2)	27.5 (5.6)	x	x		x	x	x	
24	Bolink	2015		OC		40HK	53	64.7 (8.9)	28.7 (6.1)		x		x			
25	Bolink	2015	S			20K	65	67.4 (7.7)			x	x				
26	Bolink	2016	S			36H	50	63.9 (9.8)	26.3 (3.5)			x	x			
27	Bolink	2019	S			77H	52	65 (11)	27.0 (5)			x				
28	Bolink	2012		OC		20H	65	67.4 (7.7)			x		x			
29	Calliess	2014	S			6K	50	60.2	26.1			x		x		
30	Chen	2016		OC	V	14H	79	57.2	25.0				x	x		
31	Chopra	2019		OC		10A		65.8	27.6		x		x			
32	Chopra	2017	S			24A					x	x	x	x	x	
33	Chopra	2014		A		24A	46	64.6 (6.9)	27.7 (4.7)			x	x	x		
34	Christiansen	2015	S			24K	54	65.2 (9.2)	28.9			x	x	x		
35	Clermont	2016		OC		15K		64.6 (6.8)	30.6 (4)				x			
36	De Brabandere	2020			V	20H					x			x	x	
37	De Vroey	2018			V	16K		64.1 (7.5)	32.2			x			x	
38	Fransen	2019	S			65K	54	65.0	30.0			x			x	
39	Grip	2019		OC/OS		15H	0	51.8 (9)	27.4 (3.2)			x	x	x		
40	Hafer	2020		OC	V	9K	44	69.2 (4.5)	26.2				x			
41	He	2019		A		6K	100	60.8 (1.1)	27.4 (0.6)							
42	Hiyama	2015	S			43K	81	72.0 (6.6)	25.9 (3.3)			x		x		
43	Hiyama	2020	S			27K	85	71.0 (6)	25.9 (3)			x				
44	Iijima	2019		A		131K	72	74.2 (5.8)	21.7 (2.5)					x		
45	Ishii	2020		OS		44K	50	68.9 (9.3)	25.1 (3.1)	x	x					
46	Ismailidis	2020		A		23K	48	66.1 (8.9)	28.1 (3.8)		x		x			
47	Item-Glatthorn	2012			V	26H	0	54.0 (9)	27.1					x		
48	Khan	2013		OC		38K	42				x	x	x			
49	Kluge	2018	S			24K	67	64.0 (11)	31.3 (6.8)			x	x			
50	Kobsar	2018	I			8K	50	58.0 (5)	25.3 (4.8)	x				x		
51	Kobsar	2017	I			39K		59.0 (8)	26.6 (3.8)	x				x	x	
52	Kobsar	2016			R	10K	40	57.0 (8)	26 (4.5)	x						
53	Kwasnicki	2015	S			14K	57	69.3 (4.6)	29.2 (2.8)			x	x	x		
54	L'Hermette	2008		OC	V	5H	0	72.3 (9.5)			x		x			
55	Liikavainio	2010		OC		54K	0	59.0 (5.3)	29.7 (4.7)				x			
56	Lyytinen	2016		OC/A	R	9K	0	62.7 (5.1)	27.0 (4.2)				x	x		
57	Mariani	2013		OC	V	34A	24	63.8 (17)	28.1		x	x	x			
58	Mccarthy	2013		OC		23K	61	65.1 (7.7)	28.7 (3.7)	x			x	x		
59	Na	2019		OC		26K	62	66.0 (6.1)	30.6 (5.6)	x	x		x			
60	Na	2020		OC	V	26K	62	65.9 (6.1)	30.5 (5.6)				x			
61	Nelms	2020	S			69H	47	61.2 (8.1)	26.8 (4.9)		x	x	x			
62	Odonkor	2020		OC/A		10K	60	63.9 (8.1)	33.2 (8.4)		x		x			
63	Oka	2019		A		41K	100	72.3 (7.1)	26 (3.9)					x		
64	Rahman	2015		OC/OS/A		45K	57	66.9 (10.7)	29.9 (4.7)		x	x	x	x		
65	Rapp	2015	I			29H	48	67.8 (6.3)	24.9 (4.9)		x	x	x	x		
66	Reh	2019	I			20H	20	63.0 (8.6)	27.5			x	x	x		
67	Reininga	2011			V	15H		61.0 (9)	25.6		x			x		
68	Reininga	2012		OC/OS		60H	75	59.7 (8.7)	26.6		x		x	x		
69	Rouhani	2012		OC/A		35A	26	63.5 (18.6)	28.1				x		x	
70	Rouhani	2014		OC	V	12A	33	58.0 (13)	28.4		x		x		x	
71	Rouhani	2012		OC	V	15A	26	53.3	28.0				x			
72	Saida	2020	S			18K	67	72.0 (9)	25.9 (2)			x				
73	Samani	2020		OC		19K	47	66.2 (5.2)	28.1 (2.7)	x	x		x			
74	Senden	2011	S			24K	54	70.0 (8)	27.3 (4)			x	x			
75	Staab	2014		OC	V	12K	17	44.4 (7.6)	26.9 (3.2)		x		x	x		
76	Suh	2019		A		195K	84	72.6 (6.1)	26.0 (3.1)			x				
77	Sun	2017		OC	V	23K		69.9 (6.6)	26.6 (3)		x		x			
78	Tadano	2016		OC/OS		10K		68.7 (4.1)	23.5 (2.5)		x		x			
79	Tanimoto	2017		OC/OS		12K	83	73.0	23.4 (2.5)				x			
80	Teufl	2019		OC		20H	65	56.9 (8.2)	27.4			x	x	x		
81	Turcot	2008		OC	V	9K	67	63.4 (4.6)	32.2	x	x		x	x		
82	Turcot	2008			V	25K	76	63.9 (7.6)	31.6					x		
83	van den Noort	2013		OC	V	14K	79	61.0 (9.2)	30.4					x		
84	van Hemert	2009		OS		53K		71.9 (8.3)	28.3 (3.9)			x		x		
85	Wada	2019	S			23H	100	61.0 (7.1)	23.0 (3.2)			x	x			
86	Wang	2020		OC	V	78K	57	59.7 (7.1)	23.0 (3.8)				x			
87	Youn	2018		A		18K	50	66.5 (7.7)	29.5 (4.9)			x		x		
88	Zhang	2016	S			12K	58	65.3 (8)	26.6 (3.5)			x	x	x		
89	Zijlstra	2008		OC		4K	50					x	x			
90	Zügner	2019			V	49H	49	73.0	28.7			x		x		

Abbreviations: i) Study design: Long=Longitudinal, S=Surgery, I=Other Interventions, OS=OA severity comparison, OC= OA vs. control, A=Association between outcomes, V=Validation, R=Reliability, ii) Sample: H=hip, K=knee, A=ankle, TJA=Total Joint Arthroplasty
