# Supplementary material for: Wearable Inertial Sensors for Gait Analysis in Adults with Osteoarthritis—A Scoping Review
Source: Sensors (Basel). 2020 Dec 13;20(24):7143. doi: 10.3390/s20247143 (PMC7763184; doi:10.3390/s20247143)
Supplement: Supplementary file 1 [file sensors-20-07143-s001.zip › Supplementary Materials/Supplementary_Table2.pdf]

Supplementary Table 2. Additional details on inertial sensors, protocols, and biomechanical variables for all 72 included studies.

Study Information			Sensors Specifications				Sensor Placements					Protocol		Biomechanical Variables						
Ref	Author	Year	Sensor	Range (+/-g)	Freq. (Hz)	No. Sensors	Head	Back	Thigh	Shank	Foot	Setting	Walk Length	ST	Joint Angle	Segment Angle	Joint Moment	ACC	Gyro	Machine Learning
19	Aminian	1999	IC sensors 3021 + Physilog	± 5	60	2(b)			x			IL	70m	M/S						
20	Aminian	2004	Physilog		200	4(b)			x	x		IL	10m	M	x	x				
21	Andrade	2017	L3G4200D, LSM303DLM	±16	50	2(b)		x				IL	60s	M/V				M/V	M	
22	Auvinet	1999	Locométrix		50	1(u)		x				OL	40m	M				V/S		
23	Barrois	2016	XSens MTw	+/-16	100	4(b)	x	x			x	OL	20m					M	M	
24	Bolink	2015	MicroStrain Inertia-Link		100	1(u)		x				OL	20m	M/V/S		x				
25	Bolink	2015	MicroStrain Inertia-Link		100	1(u)		x				OL	20m	M/V/S		x		M		
26	Bolink	2016	MicroStrain Inertia-Link	±5	100	1(u)		x				OL	20m	M/V/S		x				
27	Bolink	2019	MicroStrain Inertia-Link	±5	100	1(u)		x				IL	20m	M/V/S		x				
28	Bolink	2012	MicroStrain Inertia-Link	±5	100	1(u)		x				OL	20m	M/V/S		x		M		
29	Calliess	2014	Shimmer2R			3(u)		x	x	x		IL	100m	M	x					
30	Chen	2016	Smart Shoe System		50	2(b)					x	IL	15m	M				M		x
31	Chopra	2019	Physilog		200	5				x	x	OL	50m	M/S	x	x				
32	Chopra	2017	Physilog		200	6(b)				x	x	OL	50m		x	x				
33	Chopra	2014	Physilog			5				x	x	OL	50m	M/S	x	x				
34	Christiansen	2015	Delsys	± 10	1000	2(b)				x		OL	6m					M/S		
35	Clermont	2016	GENEActiv		100	1(u)		x				OL	600s	M/V						
36	De Brabandere	2020	Samsung Galaxy J5 2017		50	1(u)			x			IL					x			x
37	De Vroey	2018	Xsens MTw		100	2(u)				x		OL	6	M						
38	Fransen	2019	Dynaport Hybrid system	±6	100	1(u)		x				OL	50m	M/V				F/V/S		
39	Grip	2019	MoLabTM	±10	128	5(b)		x	x	x		OL	9m		x					
40	Hafer	2020	Opal, APDM Inc.	±16	128	4(u)		x	x	x	x	IL	10m	M	x					
41	He	2019	MPU-9250		200	1(u)						IL	10m			x	x			x
42	Hiyama	2015	MVP-RF8-HC		500	1(u)					x	IL	10	M/V						
43	Hiyama	2020	MVP-RF8-AC		500	2(u)		x			x	IL	10m	M				S		
44	Iijima	2019	TSND151		200	1(u)		x				IL	20m					S		
45	Ishii	2020	WAA-010		100	2(u)				x	x	IL	20m					M		
46	Ismailidis	2020	RehaGait			7(b)		x	x	x	x	IL	20m	M	x					
47	Item-Glatthorn	2013	IDEEA		32	5		x	x	x	x	IL		M						
48	Khan	2013	GLI Interactive LLC	± 2	100	1(u)				x		IL						M		
49	Kluge	2018	Shimmer3	±8	102.4	2(b)					x	IL	40m	M		x				x
50	Kobsar	2018	iNEMO inertial module	±16	100	4(u)		x	x	x	x	IL	150s					M		x
51	Kobsar	2017	iNEMO inertial module	±16	100	4(u)		x	x	x	x	IL	60s					M		x
52	Kobsar	2016	iNEMO inertial module	±16	100	4(u)		x	x	x	x	IL	60s					M		
53	Kwasnicki	2015	ADXL330 e-AR	±3	50	1(u)	x					OL								x
54	L'Hermette	2008	ADXL105-EM3		100	1(u)		x				IL	50m	M		x				
55	Liikavainio	2010	Meac-x	±10	1000	3			x	x		IL/OL	10m					M		
56	Lyytinen	2016	Meac-x		1000	1(u)				x		OL	15m	M				M		
57	Mariani	2013	Physilog		200	1(u)						OL	50m	M						
58	Mccarthy	2013	gaitWALK system		102.4	4(b)			x	x		OL	20m	M	x					
59	Na	2019	Noraxon		100	5(b)		x	x	x		IL	50m					M		
60	Na	2020	Noraxon			5(b)		x	x	x		IL	10m					M		
61	Nelms	2020	BioStamp			1(u)		x				IL	14m	M/S		x				
62	Odonkor	2020	Shimmer3		102.4	2(b)					x	IL	6m	M		x			M	
63	Oka	2019	MVP-RF8-HC		200	2(b)	x	x				IL	20m	M/V				M/V		
64	Rahman	2015	GaitSmart			5(b)			x	x		OL	20m	M/S	x	x				
65	Rapp	2015	Humotion		100	1(u)		x				IL	20m	M				V/S		
66	Reh	2019	XSens MVN Awinda		60	7(u)		x	x	x	x	IL	20 min	M/V/S						
67	Reininga	2011	Xsens MTx		100	2(b)	x	x				IL/OL	33m	M		x				
68	Reninga	2012	Xsens MTx		100	2(u)	x	x				OL	25m	M		x				
69	Rouhani	2012	Physilog		200	4(u)				x	x	OL	50m		x	x				
70	Rouhani	2014	Physilog		200	4(u)				x	x	OL	100m		x	x	x			
71	Rouhani	2012	Physilog		200	3				x	x	OL	100m		x	x				
72	Saida	2020	MVP-RF8		100	3(u)		x		x		IL	10m					M/V/S		
73	Samani	2020	ST microelectronics		2000	8(u)			x	x		IL	40m					M/F		x
74	Senden	2011	Minimod		100	1(u)		x				OL	20m	M/V/S		x				
75	Staab	2014	MMA76260Q	± 2	1000	3(b)		x		x		IL	500m	M/S		x				
76	Suh	2019	G-Walk			1(u)		x				OL	8m	M						
77	Sun	2017	IDEEA3		32	7(b)		x	x	x	x	IL/OL	16m	M						
78	Tadano	2016	H-Gait systems	±4	100	7(b)		x	x	x	x	IL	7m	M	x	x				
79	Tanimoto	2017	MVP-RF8-GC-500	±2	100	1(u)				x		IL	600s	M/V					M/V	
80	Teufl	2019	XSens MTw		60	5(b)		x	x	x	x	IL	600s	M	x	x				x
81	Turcot	2008	ADXL320, ENC-03J	±5	120	4(b)			x	x		IL			x			M		
82	Turcot	2008	ADXL320, ENC-03J	± 5	120	4(b)			x	x		IL						M		
83	van den Noort	2013	Xsens MTx		50	8(b)			x	x	x	IL	10m				x			
84	van Hemert	2009	The Dynaport System			6(u)		x	x	x		IL	20	M/S		x		S		
85	Wada	2019	MVP-RF8-AC		500	2(u)		x			x	IL	10m	M/V				S		
86	Wang	2020	DA14583	±4	100	2(b)				x		IL	20m				x			x
87	Youn	2018	Noraxon		200	2(b)				x		IL	6m	M				M		
88	Zhang	2016	IDEEA3			7(b)		x	x	x	x	IL	40m	M				M		
89	Zijlstra	2008	DynaPort MiniMod		100	2(u)		x				IL	30m			x				
90	Zügner	2019	GaitSmart		102.4	6(b)		x	x	x		IL		M/S	x	x				

Abbreviations: i) Sensor Specifications: b=bilateral, u=unilateral: ii) Setting, IL=in lab, OL=out-of lab, FL=free living: iii) ST=spatiotemporal: M=mean, V=variability, S=symmetry, iv) ACC=accelerometer variables: M=magnitude, V=variability, F=frequency, S=symmetry, v) GYRO=gyroscope variables: M=magnitude, V=variability, F=frequency, S=symmetry
